# Algorithm-Based Online Software for Patients' Self-Referral to Breast Clinic as an Alternative to General Practitioner Referral Pathway

**DOI:** 10.7759/cureus.11740

**Published:** 2020-11-28

**Authors:** Ahsan Rao, Adeel Abbas Dhahri, Humayun Razzaq, Eshagh Mokhtari, Azeem Majeed, Ashraf Patel

**Affiliations:** 1 Breast Surgery, Addenbrooke's Hospital, Cambridge University Hospitals NHS Foundation Trust, London, GBR; 2 General Surgery, Princess Alexandra Hospital NHS Trust, Harlow, GBR; 3 General Surgery, Southend University Hospital, Southend-on-Sea, GBR; 4 Breast Surgery, Princess Alexandra Hospital NHS Trust, Harlow, GBR; 5 Primary Care and Public Health, Imperial College London School of Public Health, London, GBR

**Keywords:** breast clinic referral, self-referral pathway, breast symptoms in primary care

## Abstract

Introduction

The study aimed to assess the accuracy of online software in the use of self-referral to breast surgery clinics for patients with new signs and symptoms. The study also evaluated the appropriateness of GP referrals to breast clinics and evaluated patients' perceptions of an online self-referral portal to the breast clinic for the assessment of breast signs and symptoms.

Design and methods

The pilot study was divided into two phases. In the first phase, prospective questionnaire-based data was collected from patients who were referred by a GP and presented to the regional breast unit with new signs and symptoms for breast conditions, Princess Alexandra Hospital NHS Trust (May - October 2018). The questionnaire assessed the time at each stage required by the patient to have a visit at the breast unit. It also asked the patient's opinion about an online self-referral portal to the surgical clinic. They were given hypothetical scenarios to evaluate their understanding of breast conditions. In the second phase, the patients presenting to symptomatic breast clinics were provided with the iPad to fill in their medical information in the online software. The data was collected between July and October 2019. The software algorithm was based on the National Institute of Clinical Health and Excellence (NICE) guidelines for breast conditions (2015). Breast surgeons’ recommendations acted as a standard to evaluate the accuracy of GPs' referrals and software outcome for each patient.

Results

There were 80 patients (mean age 49.1 [SD: 17.7], all females) included in the first phase of the study. The most common clinical presentation was a breast lump (47.6%), followed by breast pain (26.9%) and nipple changes (7.9%). Breast surgeons considered appropriate 75.6% of the referrals made by the GP. Seventy-two percent of the patients got an urgent appointment to see their GP, and 94.8% of the patients were urgently referred by their GP to see the breast surgeon. Only 37.8% of the urgent referrals were correctly referred as urgent. Having a direct online referral system for breast conditions will be beneficial for patients was agreed by 78.4%. The majority (98.1%) of the participants answered correctly for the hypothetical questions requiring breast surgeon review. In the second phase, there were a total of 86 patients with a mean age of 43.9 (SD: 13.3). The most common presentation was breast lump (n=68, 79.1%) and other presentations included breast pain, nipple changes, and discharge. The GPs' accuracy of correct referral was 69.1%. One third (30.9%) of the referrals could have been managed in the community or as a routine review by the breast surgeon. In comparison, the online software's accuracy was 85.1% accurate (p=0.001). The accuracy for detecting patients who needed urgent breast clinic review was 100% for online software.

Conclusion

A large proportion of referrals could have been dealt with in the community or referred routinely. Patients would prefer a direct online referral system to the breast clinic. They understand red flag signs and symptoms. Online software has the potential to streamline patients for symptomatic breast clinics. It can reduce the burden on the GPs who are constantly under pressure to diagnose patients accurately and refer to the correct specialty appropriately within a short time.

## Introduction

Patients with clinical signs suggestive of breast cancer such as breast lumps, pain, skin changes, or discharge should present to their GP for a referral to be seen by a breast specialist for further review and diagnosis [1]. The current system that allows a patient to be seen by a breast specialist in the UK is typically via a GP referral pathway. The GP is the primary care provider who acts as the first point of contact for these patients [2]. They not only serve as the gatekeeper who makes the referral but also assess and screen patients initially and make appropriate referrals within a specified timeframe. This can include urgent or routine referrals. 

These referrals are made to what is known as secondary care (e.g., breast clinics), or sometimes those who show a significant risk or background of breast cancer may be referred directly to tertiary care (e.g., genetic testing facilities); however, referral to tertiary care often comes from secondary care [3]. In addition to this method, the NHS has established a breast-screening program whereby women aged 50 to 71 are invited to mammography imaging every three years under the current scheme [4]. 

National Institute of Clinical Health and Excellence (NICE) guidance has suggested that those who are deemed appropriate for referral are classed into either an urgent (cancer suspected pathway) group who should be seen by the specialist within two weeks, and a non-urgent group who will see a specialist within 6-8 weeks on average [5]. With the bulk of all referrals to a breast specialist coming from the GP, there is an increasing amount of workload and pressure on them to refer appropriately and in a timely manner [6]. Additionally, the role of the primary care physician in referring to secondary care has not led to a reduction in total healthcare costs, improvement of clinical outcomes, or a more streamlined service; hence, there remains scope for alternative approaches for patients in the community who wish and need to gain access to some specialist services [7]. Data in the current literature shows an underperformance exists in referrals to surgical specialties in volume and quality [8]. 

Clinical software has been gaining the interest of clinicians in recent years. Multiple trials are being conducted to assess its use in diagnosing and prognosis of various medical conditions and improving healthcare [9]. The study aimed to assess online software as a self-referral tool for patients with a new presentation for breast conditions directly to breast surgeons as an alternative to the GP referral system. The pilot study was conducted in two phases. In the first phase, we evaluated patients' perceptions regarding the implementation and use of an alternative self-referral pathway process. The patients were also assessed for their understanding of ‘red-flag’ signs and symptoms of breast conditions. Due to recent media and public campaigns on breast cancer awareness, the majority of patients have acquired enough knowledge of sinister signs and symptoms of cancer. This precursor knowledge is important to set up a self-referral system in the long-term. In the second phase, we acquired data on the patients using online software. It was used to assess its accuracy in correctly identifying patients who require urgent review from the breast surgeons and those that not require a visit of the breast team and can be managed in the community for benign conditions.

## Materials and methods

Phase 1

This was a single-center prospective study conducted at the Princess Alexandra Hospital NHS Trust, Harlow, Essex, UK. The data was collected from 10th May 2018 to 30th October 2018. The data was collected at the Department of Breast Surgery, St Margaret’s Hospital, Epping. All patients with the first onset of new signs or symptoms for any breast condition referred by the GP were included in the study. The referral was to a one-stop clinic where a patient is seen by the breast surgeon and gets imaging and biopsy of the lesion if required. The GP refers to the patients for breast signs and symptoms. The referral can be made urgently, in which the patient must be seen by the breast surgeon within two weeks if there is a suspicion of cancer, or routinely, within six weeks, for benign breast conditions. The appropriateness of a referral was determined according to the National Institute of Clinical Health and Excellence (NICE) guidelines for ‘Breast Cancer - recognition and referral’. If a case was referred within the urgent or routine pathway timeframe, it was analyzed if the case met any of the criteria outlined within the recommendations for referral within that respective pathway as per the guidelines and hence was deemed ‘appropriate’ or ‘not appropriate’ accordingly [1]. Patients who were followed up to manage their previous breast condition were not included in the study. The patients who had a previous breast condition that was treated earlier and now presented with new symptoms or signs indicating a new breast condition were included in the study. For example, if the patient previously had the right breast lump and was then referred for a new breast lump on the opposite side, the patient was included in the study. The ethical approval to run the survey was obtained from the local Patient Quality and Safety Department at Princess Alexandra Hospital NHS Trust. 

The designed questionnaire had four main components (Appendix 1). The breast surgeon answered the first part of the questionnaire. It included information on the time it took the patient to see the GP and then the breast team. It also asked the breast surgeon whether the referrals and their urgency were appropriate, as suggested by the GP. The second part of the questionnaire asked the patients if they had difficulty in accessing their GPs. The third part of the questionnaire inquired if the patients were interested in the idea of having a direct online referral to the breast team without seeing their GP. The fourth part of the questionnaire had four hypothetical scenarios for the patient. For each scenario, the patients were asked to give their opinion on whether the patient in the scenario required review by the breast surgeon, and if that review was urgent. The scenarios were designed such that two of them did not require breast surgical review; one needed urgent breast team review, and the other entailed routine review by breast surgery. 

Once the patients were seen by the breast surgeons and were waiting for the imaging, they were handed the questionnaire to complete. The questionnaire was collected while they were leaving the clinic. The response rate of the questionnaire was 88%. 

Phase 2

The second phase of the study is the use of online software for self-referral. The data was collected between June 2019 to September 2019. The patients who were referred by the GP and attended the new symptomatic one-stop breast clinic at St Margaret’s Hospital in Epping were included in the study and were asked to feed in their presenting complaint and past medical history in the software. The software provided them with the referral outcome, which was stored online in the dataset cloud-based library. The main outcomes from the software were: urgent referral to a breast surgeon, routine referral to a breast surgeon, managed in the community by the GP, and referral to breast specialist nurse. Hence, after assessing the patient details, the software concluded how the patient should have been managed. The stratification of the patients for the type of referral by the software was based on NICE guidelines for ‘Breast Cancer - recognition and referral’ [1]. Along with the information from software, it was also recorded if the GP made an urgent or routine referral. The breast surgeon was also asked to provide input in the data by recording if the referral was appropriate and how this patient could have been best managed and referred. The breast surgeons were blinded by the outcome provided by the software. The accuracy of the referral by the software and the GP was measured by comparing it to the outcome provided by the breast surgeon. 

Statistical methods

The data was collected in Microsoft Excel (Microsoft, USA), and SPSS software (IBM Inc., Armonk, USA) was used to perform the data analysis. The comparison of dichotomous and continuous variables was made using chi-square and t-test, respectively. For the software's and GP's accuracy for correct and appropriate referrals, sensitivity and specificity were measured. Similarly, sensitivity and specificity were calculated for patients on their correct responses for hypothetical scenarios provided to them.

## Results

**Figure 1:**
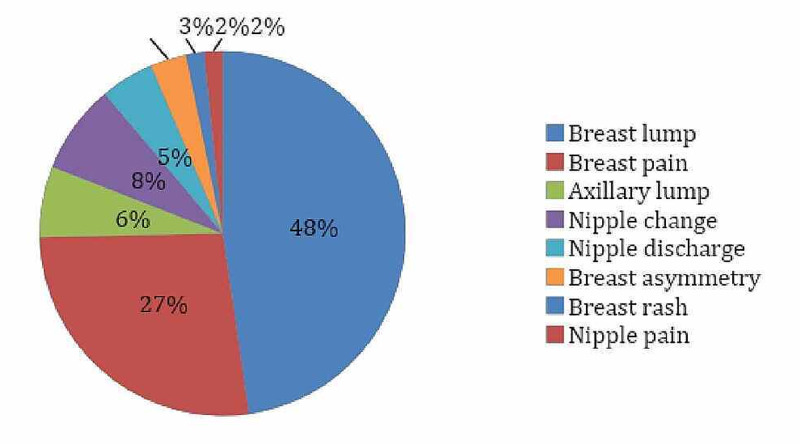
Breast signs and symptoms presented by the patients at the outpatient clinic

There were 80 patients included in the study. The patients' average age was 49.1 (SD: 17.7), and all of them were females. The most common clinical presentation was a breast lump (47.6%), followed by breast pain (26.9%) and nipple changes (7.9%) (Figure 1). The average time taken to be seen in the clinic after being referred by the GP was 10.6 days (SD 6.7). Out of all referrals made by the GP, 75.6% were considered appropriate by the breast surgeons. Out of all referrals, 24.4% were considered suitable to have been managed in the community by the GP. Out of the referrals, 37.8% were correctly referred as urgent, while the rest were inappropriately referred urgently. 

In the majority of the cases (66.6%), patients easily got an appointment from the GP for a breast sign and symptom, whereas 15.3% of the patients found it difficult to get a GP consultation. Seventy-two percent of the patients got an urgent appointment from the GP for their breast problem. The majority (94.8%) of the patients were urgently referred by their GP to see the breast surgeon within two weeks through the urgent breast cancer pathway. The majority (94.8%) of the patients were examined by their GPs before they were referred to the breast clinic. Out of all respondents, 44.3% and 34.1% of the patients agreed and strongly agreed that having a direct online referral system for breast conditions will benefit the patients, respectively. However, 34.2% of the patients still want to see their GP before being assessed by the specialist breast team. The impression of 72.2% of the patients was that online direct referral will obtain them faster review by the breast specialist. 

There were hypothetical scenarios in the questionnaire, and the patient was asked if the patient required breast specialist review, and if so, urgent or routine. The first scenario was that of a cancer lump and required urgent breast surgeon review. The second clinical situation was based on clear nipple discharge requiring routine referral, while two scenarios presented benign conditions in young patients not requiring breast specialist review. 

Overall, 98.1% of the participants answered correctly for the hypothetical patients requiring breast surgeon review; but, only 18.4% of the participants correctly answered not to have breast clinic referral for benign conditions not requiring assessment by breast specialists. For the hypothetical situation for cancer cases, all patients answered urgent breast surgeon review; hence, the sensitivity for cancer case was 100%. For potential benign conditions requiring routine breast clinic assessment, 96.2% of the patients answered to have breast specialist review for the hypothetical case, and 30% of those on a routine basis. 

There were a total of 86 patients included in the second phase of the study. The mean age of the study population was 43.9 (SD: 13.3). The most common presentation was breast lump (n=68, 79.1%) and other presentations included breast pain, nipple changes, and discharge. Only two patients were male, while the rest were females. 

In the second phase of the study, the GPs’ accuracy of correct referral was 69.1%. All the GP referrals were urgent on the breast cancer fast track referral pathway, which meant to be seen by the breast surgeon within two weeks of the referral made by the GP. One-third of the referrals (30.9%) could have been managed in the community or as a routine review by the breast surgeon. In comparison, the online software’s accuracy was 85.1% (p=0.001). However, all the patients that required urgent breast clinic review for suspicion of breast cancer were identified by the software in 100%. The main reason for inaccuracy was suggesting urgent review for the patients who either could have been managed in the community or could have seen a breast surgeon on a routine basis. It did not provide any adverse outcome for the patient who required urgent review by the breast surgeon.

## Discussion

Most of the patients urgently booked themselves to be assessed by the GPs for breast conditions, and they were further referred by the GPs to be urgently seen by the breast specialist team. The local breast team saw almost all the patients within two weeks of the referral. Most of the patients wanted to have a direct online referral pathway to the breast clinic with the impression that their breast condition will be managed better and more efficiently. The patients performed very well in hypothetical scenarios indicating that they have a reasonable understanding of breast signs and symptoms. The initial data on the AI software shows better sensitivity to identify patients who need urgent breast review and those that can be managed in the community. 

The concept of patient online referral has been tested in other specialties and has received positive feedback. In earlier studies, the online referral program was shown to be user-friendly, and the physicians' impressions were that it had detailed information to triage patients [10]. Our results were similar to a survey conducted in Iran that showed that patients would prefer to be assessed by the hospital specialist because they consider them to have more knowledge in their respected clinical areas and GP visit is time-consuming [11]. In another study, the patients who lived in an urban community, and were better educated, were the ones who opted for private care and directly contacted specialists for their medical conditions [12]. In an earlier study, it has also been shown that a self-referral pathway improved management of herpes infection in lymphoma patients when they were provided the chance to refer themselves after receiving information on herpes infections [13]. This provides an opportunity to educate patients about their conditions, sharing responsibility in the care, and empowering them for their healthcare. This will also help in patient-doctor communication, as those who self-referred themselves were more satisfied with the specialist management program and were found to be more compliant [14].

The studies have shown that patients favor direct access to specialists, but less support is received from policymakers and specialists [15]. The scare of increased referrals directly from the patient exists. The direct access option is available in some of the European countries and New Zealand, and it has not shown to have a significant increase in the number of referrals to hospital specialists [16]. The study on direct referral for physiotherapy in Scotland has shown that direct referral was more cost-effective than those patients who were referred by the GP. The overall cost of the episode care included the cost of analgesia prescribed, the number of investigations carried out, and follow up by the health services [14]. The rate of appropriate referrals was also high as compared to those referred by the GP [17].

Online clinical software and artificial intelligence technology are currently being trialed in breast surgery in different aspects of patient care. It was tested to improve breast cancer diagnosis accuracy in one of the earlier studies [18]. Similarly, it has been used to develop a prognostic tool for breast cancer [19]. In the US, the development of AI in breast cancer has used various technology types [20]. It has also used public health data to train the software and improve its validity. However, despite successful and promising results from the recent studies, it has not translated into clinical practice at a wider scale yet. In this study, the focus has been on utilizing software in improving clinical pathways for breast patients. The accuracy of the data has shown promising results and better accuracy than the GP referral pathway. 

Currently, GPs are being scrutinized for improper referrals to surgical subspecialties. This study investigated the concept of online self-referral for breast patients as an alternative to the current referral system via GP. Given the opportunity, the patients would prefer self and direct referral to specialist care. Based on hypothetical scenarios, they were aware of the red flag signs and symptoms of breast cancer. This could be due to the constant social media and public health awareness of breast cancer. The rate of accurate referral was comparable between patients who self-referred and those referred by the GP.

The data was collected from patients in the breast unit referred by the GP and did not include patients seen by the GP in primary care and not referred to the breast specialty. Although it will not assess the proportion of patients not referred to breast specialty, it will provide an evaluation of the appropriateness of referral to the specialty, which was the primary aim. The number of patients with benign conditions who were referred to the breast unit but could have been managed in the community will add to the proportion of the patients who were not referred in the first place. This will indicate what more must be considered to streamline the pathway. 

There are certain limitations of the study that must be considered for the interpretation of the results. The study has used a newly formulated questionnaire, which has not been validated. We were not able to find a validated questionnaire for the purpose of the study. The data was collected from a single-center, which could lead to potential selection bias. It only included patients who were presenting for a new sign and symptom of a breast condition. Hence, the referral of the patients with recurrent signs and symptoms must be further investigated. 

## Conclusions

In conclusion, patients with breast conditions present with high anxiety and demand urgent review by the medical team. The GP has a low threshold for the referral to the breast specialty, and most of the patients are referred inappropriately and urgently through the cancer pathway, further increasing the anxiety of the patients. Given the opportunity, the patients would prefer self and direct referral to specialist care. They also have a high tendency to refer to themselves, and they are aware of the red flag signs and symptoms of breast cancer. The online self-referral tool should be developed in a way that can then screen those patients who can be seen on a routine basis and those who can be reviewed by the GP.

## Figures and Tables

**Figure 2:**
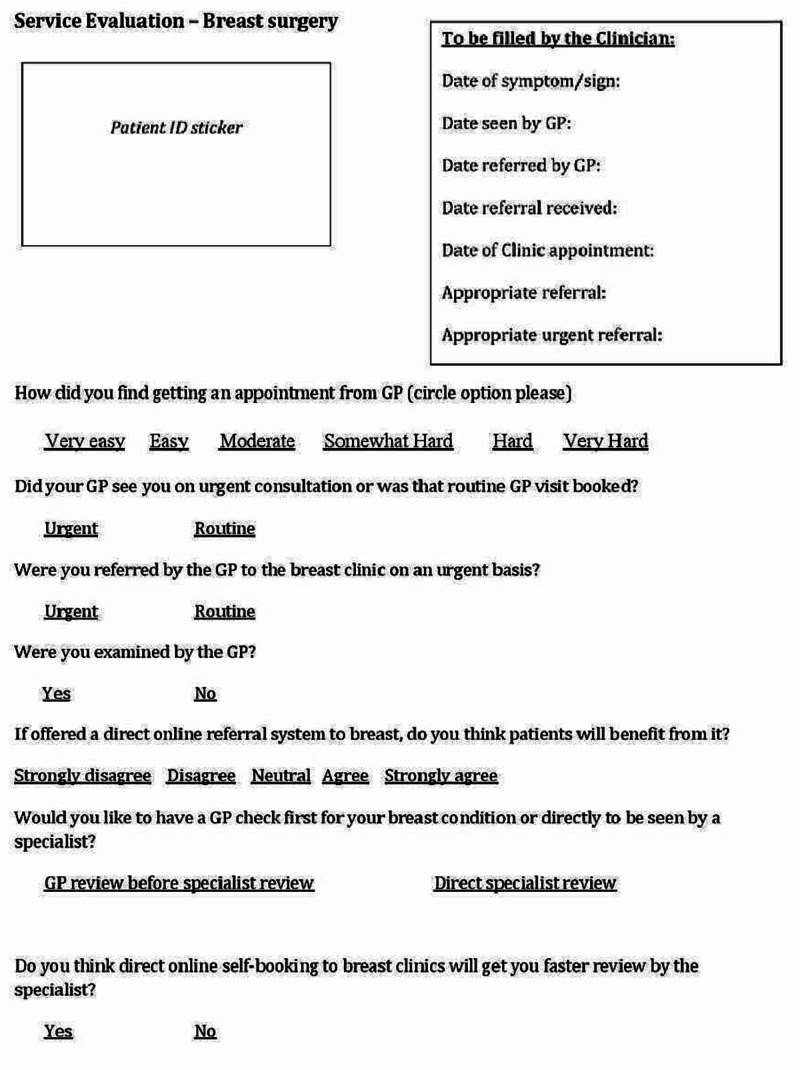
Questionnaire used in the first phase of the study

**Figure 3:**
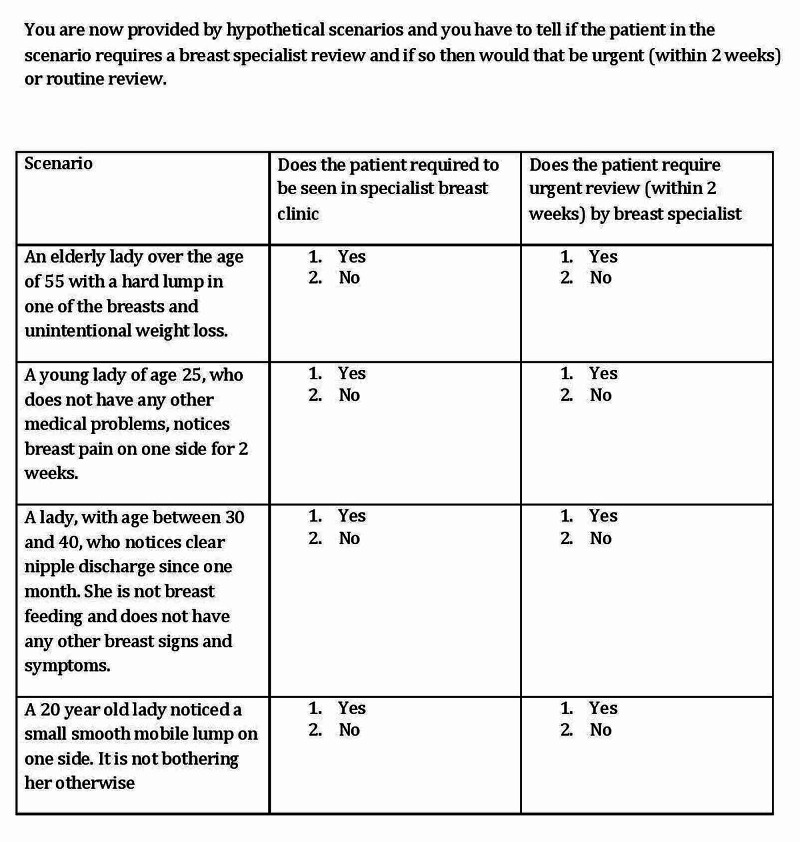
Scenarios used in the questionnaire
